# Development potential of extracellular matrix hydrogels as hemostatic materials

**DOI:** 10.3389/fbioe.2023.1187474

**Published:** 2023-06-13

**Authors:** Dan Cai, Wei Weng

**Affiliations:** Department of Orthopedics, The First People’s Hospital of Huzhou, First Affiliated Hospital of Huzhou University, Huzhou, Zhejiang, China

**Keywords:** extracellular matrix hydrogel, hemostasis, hemostatic mechanism, extracellular matrix components (ECM), hemorrhage

## Abstract

The entry of subcutaneous extracellular matrix proteins into the circulation is a key step in hemostasis initiation after vascular injury. However, in cases of severe trauma, extracellular matrix proteins are unable to cover the wound, making it difficult to effectively initiate hemostasis and resulting in a series of bleeding events. Acellular-treated extracellular matrix (ECM) hydrogels are widely used in regenerative medicine and can effectively promote tissue repair due to their high mimic nature and excellent biocompatibility. ECM hydrogels contain high concentrations of extracellular matrix proteins, including collagen, fibronectin, and laminin, which can simulate subcutaneous extracellular matrix components and participate in the hemostatic process. Therefore, it has unique advantages as a hemostatic material. This paper first reviewed the preparation, composition and structure of extracellular hydrogels, as well as their mechanical properties and safety, and then analyzed the hemostatic mechanism of the hydrogels to provide a reference for the application and research, and development of ECM hydrogels in the field of hemostasis.

## 1 Introduction

Uncontrolled bleeding is a major challenge in trauma care and surgery. Rapid and effective hemostasis is essential to improve care quality and save lives (Yang et al., 2019; Fan et al., 2021). Conventional hemostatic materials, such as tourniquets, gauze, and bandages, have shown limited efficacy in controlling bleeding (Chen et al., 2020). Moreover, gauze or bandages need to be completely removed (Leonhardt et al., 2019) after hemostasis because they are non-biodegradable, resulting in secondary injury, delayed healing, and additional pain. Therefore, there has been widespread interest in developing novel hemostatic materials and techniques. The ideal hemostatic material should have the following characteristics (Ellis-Behnke, 2011; Zhong et al., 2021): 1) the ability to quickly form thrombus; 2) It should be biocompatible, biodegradable, and conducive to accelerating wound healing; 3) stable, cost-effective, and safe.

As a novel polymer material, a hydrogel is a three-dimensional network structure with high water content (Zhang et al., 2021). The hydrogel can be applied to various irregular wounds and intraluminal injuries (Deng et al., 2017; Palomino-Durand et al., 2019) due to its injectability and fluidity, which is crucial for rapid and effective hemostasis. Furthermore, the excellent biodegradability and biocompatibility ensure the safety of hydrogel-based biomaterials for *in vivo* application and enhance their ability to promote wound healing (Labay et al., 2019). Therefore, hydrogel-based hemostatic materials have unique advantages.

Hemostasis refers to the quick stopping of bleeding (Zhang et al., 2020; D'Andrea et al., 2009; Wang et al., 2021; Zheng et al., 2020). Bleeding caused by small vessel injuries usually stops automatically within a few minutes. This phenomenon is called physiologic hemostasis. Physiologic hemostasis is one of the important protective mechanisms of the body, which include three steps: first, the injured small blood vessel immediately constricts to seal the vessel and reduce bleeding. Second, the subcutaneous matrix promotes platelet aggregation and adhesion, forming a soft hemostatic plug to fill the wound. Third, by activating the blood coagulation system, soluble fibrinogen in the plasma is converted into insoluble fibrin polymer, forming a firm mixture composed of fibrin and platelets, effectively stopping bleeding (Wang et al., 2021). However, physiological hemostasis only works in case of minor trauma (Zheng et al., 2020). Injury to an artery or viscera makes it difficult for the contracted vessels to cover the wound surface, preventing the subendothelial matrix from playing its role effectively. The subcutaneous extracellular matrix is essential for hemostasis because it contains numerous extracellular matrix proteins and can promote hemostasis in different ways (Watson, 2009; Bergmeier and Hynes, 2012; Wang et al., 2016a). Therefore, it is a novel idea to use an exogenous extracellular matrix to promote wound hemostasis when bleeding caused by trauma exceeds the ability of self-hemostasis.

**FIGURE 1:**
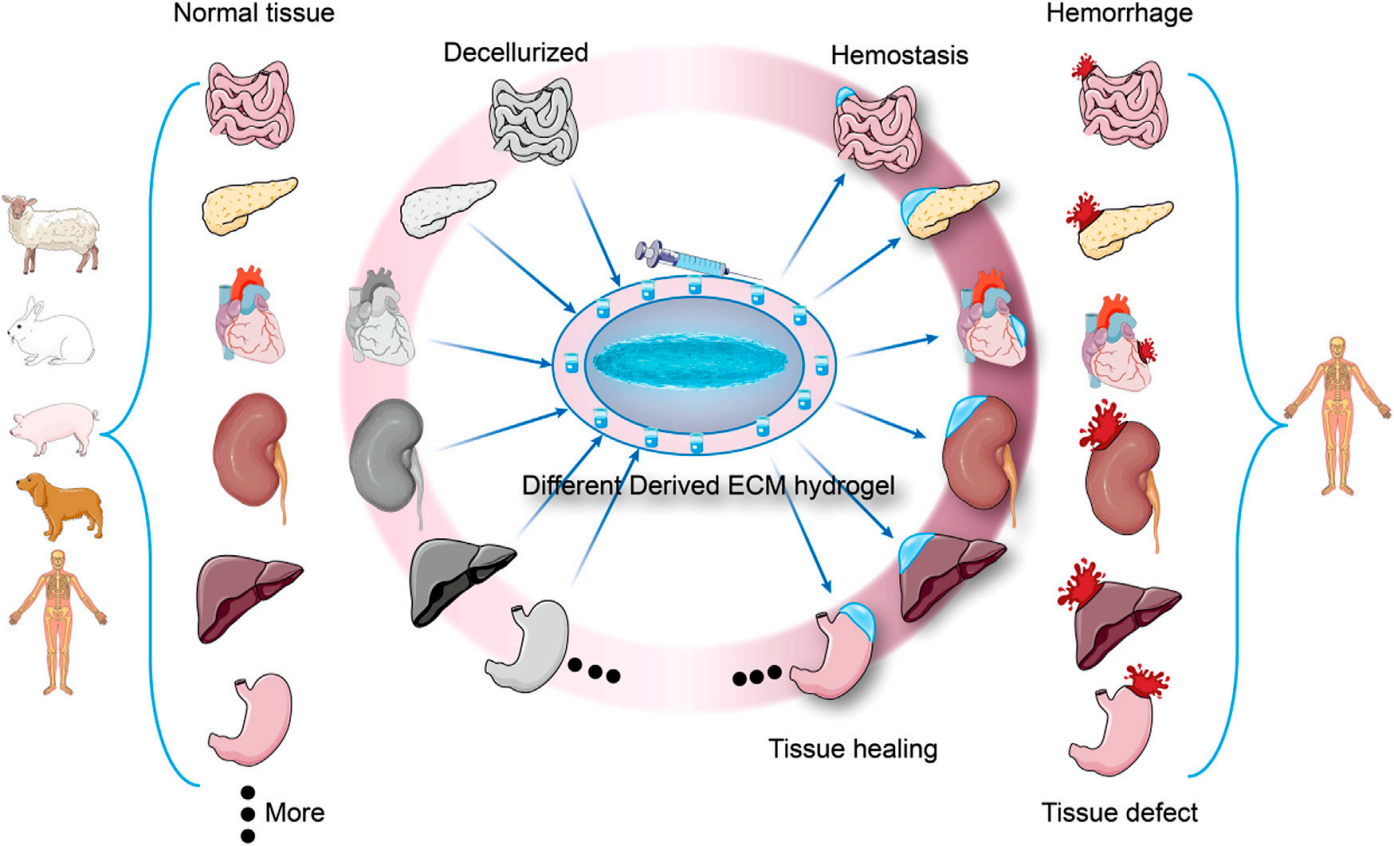
Different derived ECM hydrogels are used for hemostasis and tissue healing. Each tissue has a unique extracellular matrix structure, and when trauma results in tissue damage and bleeding, ECM hydrogels from the same tissue source can be used to stop bleeding and promote tissue healing (Specialized hemostasis and tissue healing).

ECM hydrogel has attracted much attention in the field of tissue repair and regenerative medicine in recent years due to its good cytocompatibility, biodegradability, and ability to induce tissue regeneration (Zhang et al., 2021). Native hydrogels contain a host of ECM proteins that can mimic the subcutaneous matrix to promote hemostasis. Regeneration and hemostasis can cooperate to provide tissue specific therapies. For example, When liver, kidney, or spleen tissue is injured and bleeding due to surgery or trauma, as shown in Figure 1, the corresponding ECM hydrogel of the liver, kidney, or spleen can be injected to halt bleeding and promote tissue regeneration. This review further introduces the preparation, composition, structure of ECM hydrogels, as well as their mechanical property and safety, and analyzes the role of various extracellular matrix components in hemostasis. It can provide a reference for future hemostatic material research.

## 2 Overview of extracellular matrix hydrogels

### 2.1 Source of extracellular matrix materials

The extracellular matrix can originate from cell-derived matrix, various tissues, and organs of mammals. ECM derived from *in vitro* cultured cell constructs provides a promising alternative for creating tissue engineered scaffolds (Cheng et al., 2014). For example, osteogenic ECMs can be constructed *in vitro* by culturing specific cells such as mesenchymal stem cells (Zeitouni et al., 2012), chondrocytes (Lau et al., 2012) or osteoblasts (Tour et al., 2011) under osteogenic medium. The extracellular matrix of animal origin is primarily xenogeneic, commonly derived from pig tissues and organs, but also from human cadavers and rarely from autogenic sources (Zhang et al., 2021). The sources of autologous and allogeneic tissue are minimal. Various tissues from different animals, such as the bladder (Kao et al., 2020) and heart (Seif-Naraghi et al., 2013) of pigs and the shin bone of cattle (Sawkins et al., 2013), have been widely used to overcome the shortage of human tissue to create decellularized ECM (dECM). Human-derived ECM can be sourced from cadavers, diseased or injured tissues and organs of patients, and donated tissues from human tissue banks. Obtaining autologous tissue-derived extracellular matrix requires surgical intervention (Fuller et al., 2013), decellularization, and detoxification procedures, which makes the process time-consuming.

### 2.2 Preparation of extracellular matrix hydrogels

Decellularization of biomaterials removes cellular components while maintaining the original structure, composition, biochemical, and mechanical properties of natural ECMs. Researchers have developed various decellularization methods, including physical, chemical, and enzyme treatments and combinations of these methods. For example, Sellaro et al. utilized mechanical agitation, trypsin/Ethylene diamine tetraacetic acid (EDTA), sodium deoxycholic (SDC), and Triton X-100 to create porcine liver ECM (Sellaro et al., 2010). However, each of the techniques mentioned above has its benefits and limitations. The physical method causes minimal damage to the tissue structure, but it is difficult to remove cellular components effectively (Burk et al., 2014). Chemical agents can effectively eliminate cellular components while destroying extracellular matrix proteins (Kasimir et al., 2003). Protein composition and content significantly affect the formation of extracellular matrix hydrogels. Therefore, it is crucial to consider the effect of chemical reagents on extracellular matrix proteins during the decellularization process, as shown in Table 1.

**TABLE 1:** The function and effection of acellular agents on ECM proteins in the process of decellularization.

Chemical reagent		Function	Effects on ECM proteins	Reference
Ionic detergents	SDS	Damaging cell membranes and dissociating DNA from proteins	Damaged collagen structure, reduce GAG content and growth factors	Courtman et al. (1994)
	SDC			Kasimir et al. (2003)
Non-ionic Detergents	Trition X 100		Destroyed collagen structure, increased collagen degeneration and reduce laminins/fibronectin content	Cartmell and Dunn (2000)
				Huh et al. (2018)
Acids	Hydrochloric acid	Donating hydrogen ion or form a covalent bond with an electron pair to catalyze hydrolytic degradation	Reduce collagen content	Gupta et al. (2018)
	Peracetic acid			Tsuchiya et al. (2014)
				Lin et al. (2019)
Alkalies	Ammonium hydroxide	Inducing cellular lysis by denaturation of the chromosomal DNA	Reduce GAG and collage content, eliminate ECM growth factors	Paulo Zambon, et al. (2020)
	Sodium hydroxide			Sheridan et al. (2012)
	Calcium hydroxide			Mendoza-Novelo et al. (2011)
	Sodium sulfide			Brown et al. (2011)
				Simões et al. (2017)
				Sengyoku et al. (2018)
Chelators	EDTA	Bind divalent metal cations at cell-adhesion sites of the ECM causing cell and ECM dissociation	Disrupt protein-protein interactions, and denature proteins in ECM	Brown et al. (2011)
	EGTA			Loneker et al. (2016)
Enzymes	Nuclease	Cleave nucleic acids	Harmful effects on the ECM constituents such as GAG, laminin and collagen IV	Ali et al. (2019)
	Dispase	Cleave collagen Ⅳ and fibronectin		Pati et al. (2014)
	Lipase	Catalyze the hydrolysis of lipids and aids in delipidation		Rahman et al. (2018)
	Phospholipase	Hydrolyze phospholipid components of cells and solubilizes cells		Yang et al. (2018)
	Trypsin	Cleave arginine and lysine		Meder et al. (2021)
				Kuljanin et al. (2017)
				Chirco et al. (2017)

Abbreviations: SDS, sodium dodecyl sulfate; SDC, Sodium deoxycholate; EDTA, ethylene diamine tetraacetic acid; EGTA, ethylene glycol tetraacetic acid.

The formation of extracellular matrix-derived hydrogels after decellularization is based on the self-assembly of collagen and is influenced by glycosaminoglycans, proteoglycans, and various proteins (Saldin et al., 2017; Zhang et al., 2021). The powdered decellularized extracellular matrix (dECM) is first dissolved into a homogeneous solution by enzymatic hydrolysis and acid dissolution. The formation of cross-linking gel can then be induced by adjusting the temperature, pH, or addition of cross-linking agent (Uriel et al., 2008). After the ECM powder is digested into a solution with pepsin, it contains dispersed collagen, glycosaminoglycan, proteoglycan, and ECM protein monomer. Under the suitable temperature and pH conditions or the addition of cross-linking agent, intramolecular and intermolecular cross-links of the three-dimensional helical structure of collagen monomers can be generated by covalent bonding to improve the tension and stability of collagen fibers and making ECM gel solution. Small intestinal submucosa (Mao et al., 2022), bladder (Kobayashi et al., 2020), fat (Tan et al., 2017), heart (Seif-Naraghi et al., 2013), cornea (Yazdanpanah et al., 2021a), dermis (Wolf et al., 2012), central nervous system (Tukmachev et al., 2016), umbilical cord (Výborný et al., 2019) and pancreas (Sackett et al., 2018) have been used to prepare ECM hydrogels successfully.

### 2.3 Constitutive structure of ECM hydrogels

Extracellular matrix-derived hydrogels are naturally occurring substances. After acellular treatment, the extracellular matrix retains the intrinsic structural and chemical integrity of the original tissue, which consists mainly of protein and non-protein components such as collagen, elastin, fibronectin, laminin, glycosaminoglycan, and hyaluronic acid (Halper, 2021). The ECM of each tissue is produced by its resident cells through interactions with other cell types. These cells secrete molecules that develop unique tissue structures and biochemical properties, creating an ideal microenvironment for their function. Therefore, the ECM formed by different tissues and organs after acellular treatment differs in composition, structure, and content as show in Table 2. Collagen is the main component of ECM hydrogel. Davidov et al. (2021) performed a quantitative analysis of hydrogels’ composition; artery-derived hydrogels contained approximately 64% collagen, while heart muscle, pancreas, and fat-derived hydrogels contained 20% more collagen than artery-derived hydrogels, ranging from 84% to 91%. Fibronectin (FN) is a multidomain glycoprotein present in most extracellular matrices and is involved in cell adhesion, migration, metastasis, proliferation, and differentiation (Xiao et al., 2018). Laminin is a high molecular weight multifunctional protein found in the extracellular matrix. Laminin-mediated interactions are crucial for cellular architecture formation through cell adhesion, spreading, and migration (Higaki et al., 2002). The ECM is also composed of many glycosaminoglycans (GAGs) mixtures that bind growth factors and improve water retention while giving the ECM some gel-like properties. The amount of GAG left in the tissue after decellularization largely depends on the method of decellularization. For example, ion stain removers are often used during decellularization to remove GAG from the ECM (Ebrahimi Sadrabadi et al., 2021). Therefore, ECM-derived hydrogels have a very complex composition in which different components play different roles in hemostasis.

**TABLE 2:** ECM hydrogels derived from pig and human.

Tissue source	Tissue category	Collagen	sGAG	In vivostudies	*In vitro* studies	Clinical products (ECM scafford)	
						Product and References	Function
Porcine	Artery	64%	0.54% ± 0.02%	Fu et al. (2019)	Davidov et al. (2021)		
	Cardiac tissue	84%–91%	1.50% ± 0.03%	Wassenaar et al. (2016)	Davidov et al. (2021)	Prima™Plus Christ et al. (2014)	Repair of heart valves
	Mitral valve chordae	42% ± 4%	0.22%	Seif-Naraghi et al. (2013)	Jeffords et al. (2015)	Hancock®II Valfrè et al. (2006)	
	Aortic valve leaflet	23% ± 3%	0.22%		DeQuach et al. (2010)	Mosaic® Jamieson et al. (2011)	
	Mitral valve leafle	51% ± 7%	0.22%		Wu et al. (2019)	Freestyle® Khazaal et al. (2022)	
	Pancreas	95.4%	1.30% ± 0.05%	Chaimov et al. (2017)	Davidov et al. (2021)		
					Gaetani et al. (2018)		
	Adipose	84%–91%	0.60% ± 0.02%	Tan et al. (2017)	Davidov et al. (2021)		
				Lin et al. (2016)	van Dongen et al. (2019)		
				Poon et al. (2013)	Lin et al. (2016)		
	Dermal	85% ± 4%	0.11%	Wolf et al. (2012)	Wolf et al. (2012)	Permacol® Brunner et al. (2019)	Repair of anal fistulas
				Ventura et al. (2020)	Ozpinar et al. (2021)	Strattice™ Demant et al. (2022)	Used in breast reconstruction
				Cheng et al. (2010)	Ventura et al. (2020)		
					Cheng et al. (2010)		
	Urinary bladder	58% ± 3%	0.32%	Wolf et al. (2012)	Kao et al. (2020)	MatriStem® Liang et al. (2017)	Repair of vaginal prolapses
				Ghuman et al. (2018)	Freytes et al. (2008)		
				Tukmachev et al. (2016)	Kobayashi et al. (2020)		
	Endometrium	61.3%	17.3%	López-Martínez et al. (2021a)	López-Martínez et al. (2021a)		
				López-Martínez et al. (2021b)	López-Martínez et al. (2021b)		
	Cornea	72% ± 10%	20% ± 3%	Yazdanpanah et al. (2021a)	Yazdanpanah et al. (2021b)		
				Wang et al. (2020)	Fernández-Pérez and Ahearne (2019)		
					Yazdanpanah et al. (2021a)		
					Wang et al. (2020)		
	Brain	53.75% ± 2.69%	0.51% ± 0.14%	No reported	Medberry et al. (2013)		
					Simsa et al. (2021)		
					Seo et al. (2020)		
	Spinal cord	70.32% ± 4.73%	0.13% ± 0.09%	Tukmachev et al. (2016)	Medberry et al. (2013)		
	Intestinal submucosa	51%	0.39% ± 0.03%	Wang et al. (2016b) (Okada et al. (2010) (Mao et al. (2022) Kim and Kim, (2016)	Kobayashi et al. (2020) Kellaway et al. (2023) Kim et al. (2022) Crapo and Wnag, (2010) Wang et al. (2019)	Oasis® Holmes et al. (2013)	Repair of skin wounds
						CuffPatch® Barber et al. (2006)	Repair of tendon tears
	Skeletal muscle	64.8% ± 6.9%	1.67% ± 0.01%	Zhang et al. (2018)	Zhang et al. (2018)		
					DeQuach et al. (2010)		
					Ungerleider et al. (2015)		
					Fu et al. (2016)		
Human	Umbilical cord	54.3%	0.66%	Výborný et al. (2019)	Výborný et al. (2019)		
					Ramzan et al. (2022)		
	Cardiac tissue	71.55%	0.08%–0.96%	Johnson et al. (2014)	Johnson et al. (2014)	IOPatch™ Chen and Liu, (2016)	Ophthalmologic repair
					Johnson et al. (2016)		
	Pancreas	7.54% ± 1.68%	15.2%	Sackett et al. (2018)	Sackett et al. (2018)		
					Peloso et al. (2016)		
					Tremmel et al. (2022)		
	Adipose	72% ± 4%	0.23% ± 0.05%	Chen et al. (2021)	Chen et al. (2021)		
				Zhao et al. (2019)	Zhao et al. (2019)		
				Adam Young et al. (2014)	Getova et al. (2019)		

### 2.4 The safety of ECM hydrogels

ECM hydrogels are widely used as scaffold materials in regenerative medicine due to their unique biological activity and good biocompatibility (Saldin et al., 2017; Zhang et al., 2021). ECM components are less vulnerable to rejection because their structure and function are highly conserved and nearly identical across species. ECM hydrogels, which eliminate many cell components compared to allograft and xenograft, can effectively reduce the potential for adverse host reactions after implantation. When compared to other synthetic polymers, ECM components of hydrogels are retained after decellularization, providing a better microenvironment for cell attachment and cell-ECM interaction (Badylak and Gilbert, 2008; Saldin et al., 2017).

ECM scaffolds are manufactured using various tissues derived from cells, animals, or humans. The dECM scaffold derived from cells has several advantages. For example, cultured cells can be screened for pathogens and then kept free of pathogens for ECM. In addition, after acellular treatment, the cell-derived matrix has improved plasticity and optimal porosity due to its loose structure (Zhu et al., 2021). Importantly, they can generate autologous ECM scaffolds from autologous cells, thereby avoiding the adverse host reactions induced by allogeneic or heterogeneous materials and circumventing the limited availability of autologous tissue. However, cell-derived dECM typically has limited mechanical properties (Guan et al., 2022).

Pigs are the primary source of animal extracellular matrix components. Compared with other animals, porcine organs are readily available in larger quantities and are comparable in size and function to human organs. Therefore, pigs have always been the preferred source of cellular scaffolds of tissues and organs (Zhang et al., 2021). Many commercialized porcine extracellular matrix products (Prima™ Plus, Hancock^®^ II, Mosaic^®^, Freestyle^®^, Permacol^®^, Strattice™, MatriStem^®^, Oasis^®^, and CuffPatch^®^) (Table 2) are employed in tissue regeneration. For example, Oasis^®^, derived from acellular porcine small intestinal submucosa, is an acellular product primarily used in the treatment of chronic wounds (Holmes et al., 2013). Extracellular matrix hydrogels are prepared based on extracellular matrix scaffolds. Traverse et al. (2019) conducted a first-in-man, single-arm, multicenter trial to demonstrate the safety, feasibility, and preliminary efficacy of percutaneous trans-endocardial delivery of VentriGel (an extracellular matrix hydrogel derived from decellularized porcine myocardium) in early and late MI (Myocardial infarction) patients with left ventricular (LV) dysfunction, which is the first demonstration of using a decellularized ECM hydrogel in any tissue in patients. Interestingly, ECM hydrogel can be used as an embolic agent to embolize arteries and promote vascular healing. Animal experiments have shown no signs of lymphadenopathy, pulmonary emboli, or stroke, suggesting that ECM-based nanocomposite hydrogel was safe even when used in blood vessels (Hu et al., 2020). Porcine endogenous retroviruses (PERV) are present in the pig genome and could pose a safety hazard (Kimsa et al., 2014). However, the risk is minimal because the source pigs will be housed in specific pathogen-free, biosecure conditions and are regularly monitored (Cooper et al., 2018).

Various human tissues and organs, such as cardiac tissue (Johnson et al., 2014), pancreas (Sackett et al., 2018), and adipose (Chen et al., 2021), are utilized to produce dECM. Gao et al. developed a human cardiac tissue-derived scaffold using decellularization, which improved the functional behavior of cardiac progenitor cells from patients with congenital heart disease, including cell adhesion, survival, and proliferation (Gao et al., 2022). Human-derived ECM materials are not controllable and are easily affected by donor age, degree of damage, and storage period (Johnson et al., 2014). However, those materials can effectively prevent the transmission of xenogenetic diseases.

## 3 Hemostatic mechanism of hydrogel

### 3.1 Physical barrier

**FIGURE 2:**
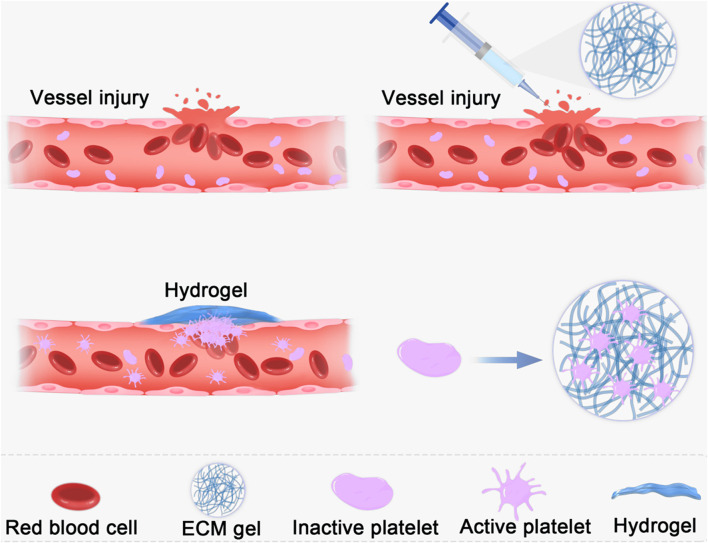
Schematic diagram of the use of ECM hydrogel showed irregular fibrous reticular scaffolds that mechanically sealed the vascular breach and formed a physical barrier.

Temperature-sensitive hydrogels are of interest for achieving effective hemostasis and wound closure because they are suitable for wounds of various shapes (Liu et al., 2018; Cao et al., 2020). ECM hydrogels have good temperature sensitivity, existing in a liquid state at 4°C and a gel state at 37°C. Fully gelated ECMs usually appear as irregular nanofiber scaffolds with interconnected pores on SEM images (Freytes et al., 2008; Wolf et al., 2012). The thermal characteristics of hydrogels can be used to stop bleeding in wounds, especially irregular wounds. The liquid hydrogel can cover irregular wounds at low temperatures; when the temperature rises to body temperature, the hydrogel transforms into a gel. The microstate showed irregular fibrous reticular scaffolds that mechanically sealed the vascular breach and formed a physical barrier as shown in Figure 2. The hydrogel is injectable and can be injected into deep tissue wounds for hemostasis (Pourshahrestani et al., 2020). Compared to natural tissues, hydrogels exhibit poor mechanical properties (Grover et al., 2014; Ahearne and Coyle, 2016), making them susceptible to deformation. Increasing hydrogel mechanical strength can effectively facilitate wound sealing and prevent blood loss. There are two main methods to improve the mechanical properties of ECM hydrogel. The first approach is to increase the initial concentration of the extracellular matrix. The second approach is to use cross-linking techniques to improve the mechanical properties of hydrogels.

#### 3.1.1 Effect of concentration on mechanical properties

Extracellular matrix hydrogels have different mechanical properties closely connected to their composition. Davidov et al. compared the mechanical properties of ECM hydrogels derived from the porcine liver, pancreas, artery, and heart (Davidov et al., 2021). It was found that arterial hydrogels exhibit the highest mechanical properties, while pancreatic hydrogels exhibit the lowest. The mechanical properties of hydrogels vary with the species of origin. The porcine myocardial matrix is significantly higher than the Human myocardial matrix by measuring storage and loss modulus with a parallel plate rheometer (Johnson et al., 2014). The mechanical properties of hydrogels vary with the species of origin Matrix, and mechanical properties can be effectively enhanced by increasing the concentration of its extracellular matrix. Tissue fibrosis typically increases various extracellular matrix components. Normal heart tissue has an elastic modulus of 10–15 kPa, while fibrotic tissue can be 2 to 10 times stiffer. Healthy lung tissue is relatively soft, ranging from 1 to 5 kPa, and can stiffen above 10 kPa in pulmonary fibrosis (Hewawasam et al., 2023).

Hydrogels prepared with a high concentration of extracellular matrix typically have greater mechanical strength. Medberry et al. (2013) prepared brain-ECM, spinal cord-ECM, and urinary bladder-ECM hydrogels. As the temperature rapidly increased from 10°C to 37°C, the maximum storage modulus, maximum loss modulus, and the time to complete gelation increased for the three hydrogels with increasing ECM concentration. Dermal-ECM hydrogels were evaluated for their structural, mechanical, and *in vitro* cell response characteristics, which were found to depend on the ECM concentration (Wolf et al., 2012). These findings suggest that the ECM concentration can influence and control the physical properties of an ECM hydrogel.

#### 3.1.2 Effect of crosslinking technology on mechanical properties

The application of crosslinking agents has been investigated to enhance the mechanical properties of hydrogels (Pilipchuk et al., 2013; Ahearne and Coyle, 2016; Parthiban et al., 2021). Crosslinking agents are introduced to modify various biomaterials and improve their mechanical properties by considering their composition and structural characteristics. Cell compatibility is a crucial evaluation factor, for example, using glutaraldehyde (GA) as a crosslinking agent might cause cell toxicity (Wang et al., 2014). Therefore, it is vital to consider the reaction of crosslinking agents on cells while enhancing mechanical features, as shown in Table 3.

**TABLE 3:** Application of crosslinking agents in ECM hydrogels.

Tissue source	Crosslinking mode	Function	Cytocompatibility	Reference
Human derived dentin matrix	dECM strongly interacted with the GelMA matrix via covalent interactions between aldehyde in dECM and amine groups in GelMA	The compressive strength improved 2-fold with increasing dECM content from 2.5 wt% to 10 wt%	Hydrogels showed a tendency to increase cell viability with the increase of dECM concentration	Sadeghian et al. (2023)
Human derived bone matrix	Demineralized and decellularized bone matrix was functionalized with methacrylate group to form photocrosslinked methacrylate bone ECM hydrogel	The mechanical properties of BoneMA were tunable, with the elastic modulus increasing as a function of photocrosslinking time	Hydrogels supported vascularization of endothelial cells and within a day led to the formation of interconnected vascular networks	Parthiban et al. (2021)
Rat derived dermal matrix	The dermal extracellular matrix hydrogel was prepared and covalently cross-linked by glutaraldehyde (GA)	Compression tests indicated that elastic moduli and yield stress values increased signifificantly with GA exposure time	Hydrogels supported cell adhesion and showed good tolerance *in vivo*	Pilipchuk et al. (2013)
porcine cornea matrix	The cornea, liver and heart extracellular matrix hydrogel was prepared respectively and covalently cross-linked by UVA-riboflavin	It can be used to enhance the mechanical properties of ECM Hydrogels. The stiffness can be controlled by varying the UVA exposure time	Hydrogels did not have any significant adverse effects on cell viability	Ahearne and Coyle (2016)
ovine liver matrix				
ovine heart matrix				
Porcine myocardial matrix	Cross-linking the ECM proteins with an amine-reactive PEG-star	Addition of PEG to the myocardial matrix did increase the stiffness of the hydrogels, although this was greater with the radical polymerization with the four-armed PEG	Hydrogels did not prevent cell adhesion and migration through the hydrogels	Wang et al. (2014)
	Myocardial matrix, PEG-acrylate, and Irgacure 2,959 were mixedand Gel formation photo-induced radical polymerization			
	Myocardial matrix, PEG-diacrylate, and Irgacure 2,959 were mixed and Gel formation photo-induced radical polymerization			
	Crosslinking of the myocardial matrix was induced during self-assembly, through the addition of glutaraldehyde (GA)	Crosslinking increases the stiffness and elasticity of the hydrogel, as assessed by parallel plate rheology	Migration of cells through crosslinked gels was slowed, but not inhibited	Singelyn and Christman (2011)
human cartilage matrix	The forming hydrogels were composed of different ionic crosslinked alginate concentrations with 1% w/v enzymatically crosslinked phenolized cartilage ECM, resulting in an interpenetrating polymer network (IPN)	The results demonstrated that upon increasing the alginate concentration, the compression modulus improved	Hydrogels provide a suitable microenvironment for the growth and viability of Human primary chondrocyte cells	Shojarazavi et al. (2021)

Abbreviations: BG, bioactive glass; GelMA, gelatin methacrylate; BoneMA, a photocrosslinkable methacrylate bone ECM, hydrogel-bone-derived biomaterial; PEG-star, Four-arm polyethylene glycol. UVA, Ultraviolet Radiation A.

### 3.2 Simulate physiologic hemostasis

**FIGURE 3:**
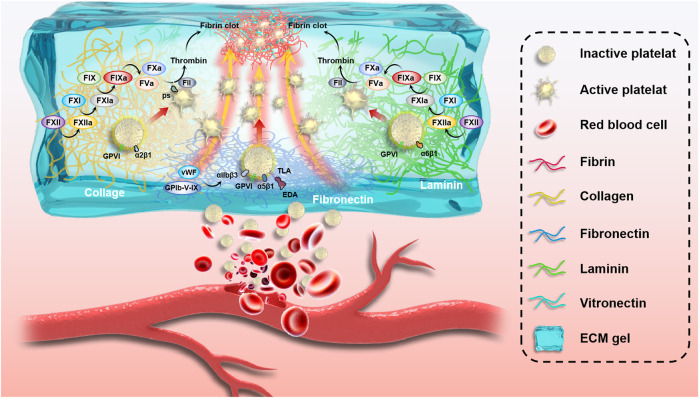
Schematic of the complex mechanism of blood vessel hemostasis. Vessel wall rupture makes it difficult for ECM proteins to play a hemostatic role, resulting in a series of bleeding events and even death. Exogenous ECM hydrogels mimic extracellular matrix main ingredients, including collagen, laminin, fibronectin, and vitronectin, which induce platelet adhesion and activation and promote hemostasis and thrombosis.

**FIGURE 4:**
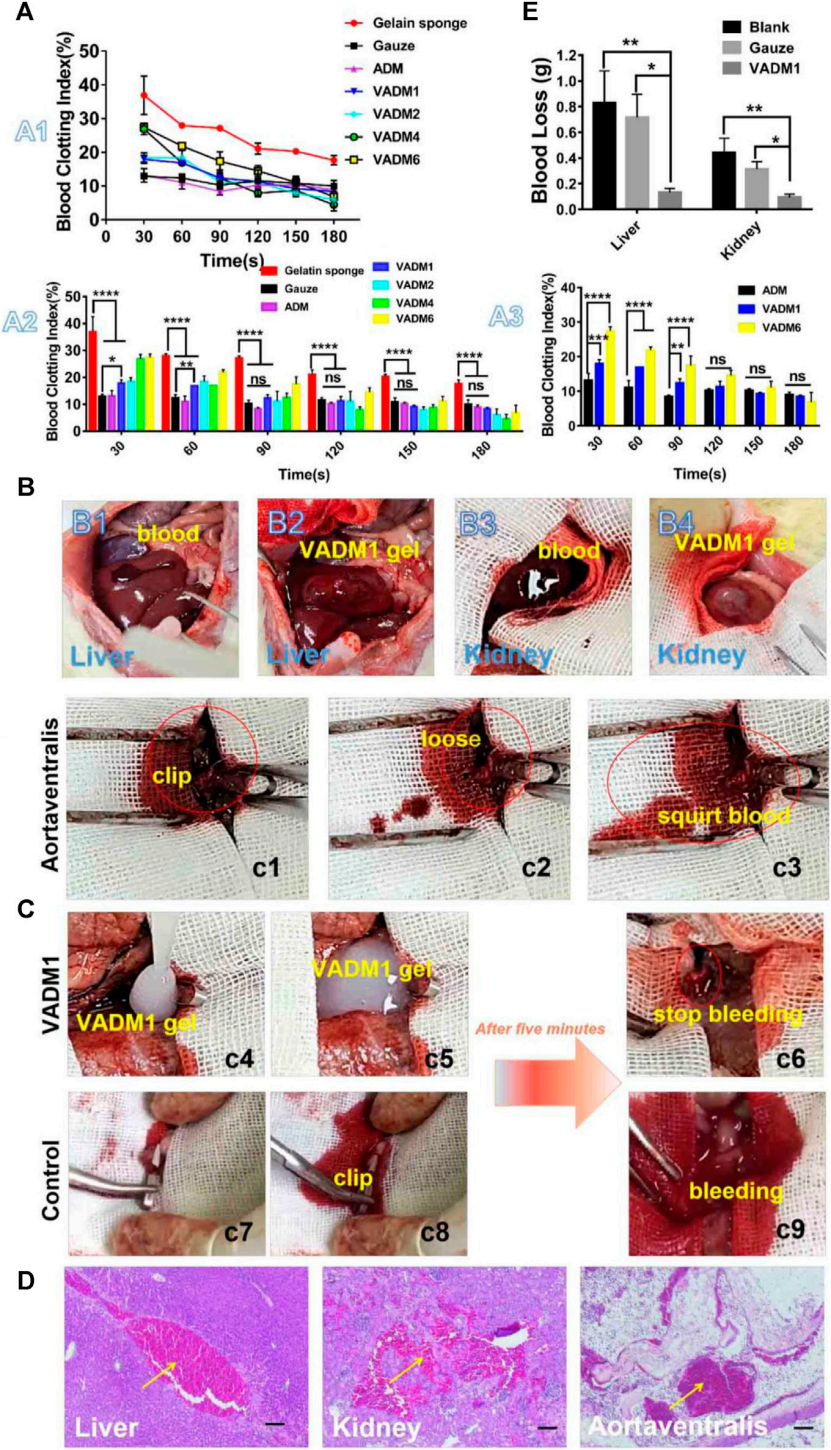
*In vitro* and *in vivo* hemostasis test. **(A)** *In vitro* dynamic whole-blood clotting evaluation (A1, A2, and A3) of the ADM, VADM1, VADM2, VADM4, and VADM6 hydrogels, with gelatin sponge and gauze used as the control. **(B)** Photographs captured during the liver laceration model and renal tissue-defect model. (B1) A wound with a length of 1 cm in the liver. (B2) VADM1 hydrogel formed *in situ* on the trauma injury. (B3) A small portion of the kidney tissue was removed with surgical scissors. (B4) VADM1 hydrogel formed *in situ* on the defective tissue. **(C)** Photographs captured during a hemostasis test on the abdominal aorta (highlighted by red circles and ellipses). (c1) Clipped abdominal aorta. (c2,c3) Arterial spurts can be observed after release of the artery clip. (c4) VADM1 hydrogel applied to the wound. (c5) Abdominal aorta surrounded by VADM1 hydrogel. (c6) Artery clip was removed after 5 min, and the bleeding was stopped completely. The abdominal aorta was clogged with hydrogel. (c7–c9) Arterial spurts still occur on the control group after 5 min, when the artery clip was released. **(D)** H&E stained micrographs, showing signifificant accumulation of red cells (yellow arrow) within the incision site. Scale bar ¼ 100 μm. **(E)** Blood loss from liver and kidney incisions. Three replicates of each sample were tested and the data were shown as mean ± SD. VADM:acellular dermal matrix hydrogel blended with vancomycin. Reprinted from (Cai et al., 2021) with permission from Elsevier Publisher, Ltd.

The inner wall of most blood vessels is covered by a continuous layer of endothelial cells that seals the subcutaneous extracellular matrix components and provides an anti-thrombotic surface for the body. Moreover, it actively secretes platelet activation inhibitors, such as nitric oxide and prostacyclin (Hein et al., 2009). Which regulate blood circulation and prevent thrombosis (Gimbrone et al., 2000). However, in the case of trauma, the ruptured vascular wall is difficult to effectively play the role of hemostasis, leading to a series of bleeding and even death events. Exogenous ECM hydrogels mimic the significant components of the extracellular matrix, including collagen, laminin, fibronectin, and vitronectin, which induce platelet adhesion and activation and promote hemostasis and thrombosis. Due to the different molecular environments of the original tissue ECM, these hydrogels have varying compositions and contents, which play different roles in hemostasis (Figure 3). According to the research of Cai et al., 2021 as shown in Figure 4, extracellular matrix hydrogels derived from porcine dermal were used in liver, kidney, and vascular trauma models of Sprague-Dawley rats and the pathological section suggests thrombosis, which was found that ECM hydrogels effectively played a hemostatic role.

#### 3.2.1 The role of collagen in hemastasis

Collagen is the main component of extracellular matrix hydrogel and is also a potent platelets activator. Its hemostatic mechanism is the effect on platelets to shorten the thrombosis time. Glycoprotein VI (GPVI) and α2β1 integrin are the collagen receptors on platelets (Sarratt et al., 2005). Collagen interacts directly with platelets via GPVI, mediates platelet activation, and integrin α2β1, which supports platelet adhesion to collagen (Jarvis et al., 2008; Attwood et al., 2013). Furthermore, collagen induces exposure of procoagulant phospholipids on platelets via GPVI(Manon-Jensen et al., 2016). The exposure of phospholipids provides an assembly site for coagulation factors, resulting in thrombin production required for platelet-fibrin thrombus. Simultaneously, collagen activates the intrinsic coagulation pathway by binding to factor XII(FXII, a coagulation factor) (van der Meijden et al., 2009). Therefore, collagen can promote blood coagulation through various pathways and plays a leading role in the process of ECM hemostasis.

#### 3.2.2 The role of laminin in hemastasis

Laminin is a heterotrimeric glycoprotein found in almost all ECM tissues, especially in the basement membrane of the vasculature. Platelet recruitment by VWF (von Willebrand Factor) enables integrin α6β1 and GPVI to interact with laminin, supporting integrin activation and resulting in stable adhesion and platelet aggregates formation (Inoue et al., 2006). This mechanism of platelet adhesion and activation is similar to platelet-collagen interaction; integrin α2β1 binding to collagen facilitates the interaction between GPVI and collagen. Further studies by Inoue et al. (2008) discovered that immobilized laminin promotes platelet recruitment under shear flow in a GPIbα-vWF dependent manner. White-Adams et al. demonstrated that laminin can activate FXII and that surface-associated laminin alone can trigger fibrin- and platelet-rich clots formation under shear (White-Adams et al., 2010). Therefore, the process of platelet recruitment, activation, and adhesion on laminin is mechanically similar to the interaction between platelets and collagen under shearing, and both can activate FXII, suggesting that laminin and collagen may jointly promote hemostasis.

Laminin is a weak platelet agonist, with a 10-fold lower affinity for the interaction between laminin and GPVI than collagen binding (Watson, 2009). Because superficial injury does not expose fibrous collagen, laminin-mediated platelet adhesion activation may be more effective. When the vessel wall is severely damaged, the exposed collagen in the deeper layers of the extracellular matrix collaborates with the superficial laminin to promote hemostasis. Moreover, laminin-111 can induce fibronectin assembly after adhesion to platelets via integrin α6β1. Therefore, laminin can indirectly affect hemostasis and thrombosis by regulating fibronectin deposition in thrombus (Cho and Mosher, 2006). As an important component of extracellular matrix hydrogel, laminin can collaborate with other hydrogel components to exert a hemostatic effect.

#### 3.2.3 The role of fibronectin in hemastasis

Fibronectin (FN) is a dimeric protein composed of two approximately 250 kDa subunits that have many biological functions and is involved in cell migration, adhesion, proliferation, hemostasis, and tissue repair (Wang et al., 2008; Tong et al., 2016). FN is present as plasma Fibronectin (pFN) and cell Fibronectin (cFN) in extracellular connective tissue matrix and extracellular fluid (Roberts et al., 2020). pFN is produced by hepatocytes and endothelial cells in the liver and exists as a soluble non-complex molecule in the blood, whereas cFN is secreted and synthesized by fibroblast and mesenchymal cells and exists in the ECM as an insoluble polymer. Fibronectin is an essential component of tissue ECM, and it exists in two forms: as a soluble form in plasma and as an insoluble polymerized form. The main difference between the two forms of existence is that cFN has an extra domain A (EDA) and an extra domain B (EDB) compared to pFN(Wang et al., 2008).

The fibrillar cFN in the ECM is a strong prothrombotic surface that promotes platelet adhesion, aggregation, and coagulation. Fibrillar cFN effectively supports platelet adhesion, and adherent platelets can be activated to form a thrombus. This process depends on the integration of α5β1 and αIIbβ3, together with the GPIb-V-IX complexes, GPVI, and TLR4 (Maurer et al., 2015). Integrins α5β1 and αIIbβ3 ensure the initial phase of platelet adhesion to fibrillar cFN. Integrins can effectively promote platelet activation once platelets are attached to fibrillar cFn (McCarty et al., 2004; Lickert et al., 2022). GPIb-V-IX complex is a membrane protein component on the surface of platelets that plays a key role in platelet thrombosis initiation and coagulation. The binding of von Willebrand factor (VWF) to the platelet membrane glycoprotein (GP) Ib-IX-V complex initiates a signaling cascade that activates αIIbβ3 and causes platelet aggregation (Liu et al., 2005). As a fibronectin receptor, activated αIIbβ3 can further promote platelet adhesion and activation. Maurer et al. (2015) decreased thrombus formation on fibrillar cFN by antagonizing the binding of VWF and GPIb-V-IX complexes. GPVI is not only a collagen and laminin agonist but also a ligand and agonist for fibronectin, which, together with integrins, promotes platelet adhesion activation (Perrella et al., 2021). TLR4 is an EDA-binding receptor involved in platelet aggregation on fibrillar cFN, and the volume of thrombus on fibrillar cFN was reduced by using TLR4 blockers (Maurer et al., 2015; Prakash et al., 2015). Fibronectin can bind to various platelet receptors and play an important role in hemostasis and thrombosis.

#### 3.2.4 The role of vitronectin in hemastasis

Vitronectin (VN) is a multifunctional 75-kDa glycoprotein present in plasma, extracellular matrix, and the α-granules of platelets (Bergmeier and Hynes, 2012). Vitronectin can not only improve platelet adhesion and aggregation during thrombus formation, but it also promotes thrombus stability. Ekmekci et al. (2002) discovered high vitronectin levels in growing thrombus, suggesting that it is actively involved in thrombosis after vascular injury. Vitronectin has two fibrin binding sites that have the potential to link fibrin monomers to polymers, binding them to fibrin clots to promote further platelet adhesion and aggregation (Schvartz et al., 2002). Platelets are covered by VN after initial binding to fibrin, and VN incorporated into fibrin clots enhances platelet adhesion and aggregation via the homotypic binding of VN molecules present on platelet surfaces and in clots. Wu et al. (2004) perfused whole blood onto a fibrin network made from purified fibrinogen, resulting in approximately 20% of the surface being covered with platelets binding purified polymeric VN to the fibrin network, resulting in a 2-fold increase in platelet surface coverage and enhanced platelet aggregate formation.

Plasminogen activator inhibitor-1 (PAI-1) can prevent fibrinolysis by inhibiting the conversion of plasminogen to plasmin. VN can bind to the β-sheet-A subunit of PAI-1, stabilize its structure, inhibit its spontaneous inactivation, and prolong its role in the fibrinolytic system, reducing the fibrinolysis of thrombi (Eitzman et al., 1995; Hess et al., 1995). Multiple protein components of ECM hydrogel could effectively induce platelet adhesion and aggregation to achieve hemostasis; vitronectin further promotes thrombogenesis in platelets by binding to fibrin sites. Moreover, thrombus stability was effectively enhanced by stabilizing the PAI-I structure and inhibiting thrombus degradation.

## 4 Conclusion and future perspectives

This study discusses the manufacturing methods, composition, safety, mechanical properties, and role of extracellular matrix proteins in the hemostasis of extracellular matrix hydrogels. With continued research and development, ECM hydrogel will be the most competitive material in the field of hemostasis. ECM hydrogels have developed a mature preparation and validation system to promote tissue regeneration, including material acquisition, acellular treatment, hydrogel formation, *in vitro* cytocompatibility experiment, *in vivo* regeneration experiment, and clinical trials. These experimental and theoretical findings facilitate the investigation of ECM hydrogels for hemostasis. Although ECM hydrogel has the potential to be hemostatic, significant experimental studies are required before clinical application. The formation and hemostatic function of the hydrogel primarily depend on extracellular matrix proteins. Protein contents vary between hydrogels from different sources and tissues, and the application of acellular reagents may destroy extracellular matrix proteins. Therefore, the hemostatic effects of hydrogels from various sources and tissues can be compared to select appropriate hemostatic agents. Optimizing the decellularization method and reducing the destructive effect of chemical reagents on ECM are essential processes. Combining tissue regeneration and hemostasis will be the focus of future research on ECM hydrogel. The primary focus of research is to promote tissue healing while enhancing the hemostatic effect. Although the hemostatic effect can be improved by introducing cross-linking agent or increasing extracellular matrix protein, hydrogel’s regeneration and repair effect is easily influenced. In conclusion, extracellular matrix hydrogels have shown immense potential in the field of hemostasis. However, further research and exploration are necessary fully realize its potential.
